# A novel echocardiographic imaging technique, intracatheter echocardiography, to guide veno-venous extracorporeal membrane oxygenation cannulae placement in a validated ovine model

**DOI:** 10.1186/2197-425X-2-2

**Published:** 2014-02-06

**Authors:** David G Platts, Andrew Hilton, Sara Diab, Charles McDonald, Matthew Tunbridge, Saul Chemonges, Kimble R Dunster, Kiran Shekar, Darryl J Burstow, John F Fraser

**Affiliations:** Department of Echocardiography, Cardiac Investigations Unit, The Prince Charles Hospital, Rode Rd., Chermside, Brisbane, Queensland 4032 Australia; Critical Care Research Group, The Prince Charles Hospital, Rode Rd., Chermside, Brisbane, Queensland 4032 Australia; The University of Queensland, Brisbane, Queensland 4072 Australia; Intensive Care Unit, The Austin Hospital, Heidelberg, Melbourne, Victoria 3084 Australia; Science and Engineering Faculty, Queensland University of Technology, Brisbane, Queensland 4000 Australia; Adult Intensive Care Service, The Prince Charles Hospital, Rode Rd., Chermside, Brisbane, Queensland 4032 Australia

## Abstract

**Background:**

Echocardiography plays a fundamental role in cannulae insertion and positioning for extracorporeal membrane oxygenation (ECMO). Optimal access and return cannulae orientation is required to prevent recirculation. The aim of this study was to compare a novel imaging technique, intracatheter echocardiography (iCATHe), with conventional intracardiac echocardiography (ICE) to guide placement of ECMO access and return venous cannulae.

**Methods:**

Twenty sheep were commenced on veno-venous ECMO (VV ECMO). Access and return ECMO cannulae were positioned using an ICE-guided technique. Following the assessment of cannulae position, the ICE probe was then introduced inside the cannulae, noting location of the tip. After 24 h, the sheep were euthanized and cannulae position was determined at post mortem. The two-tailed McNemar test was used to compare iCATHe with ICE cannulae positioning.

**Results:**

ICE and iCATHe imaging was possible in all 20 sheep commenced on ECMO. There was no significant difference between the two methods in assessing access cannula position (proportion correct for each 90%, incorrect 10%). However, there was a significant difference between ICE and iCATHe success rates for the return cannula (*p* = 0.001). Proportion correct for iCATHe and ICE was 80% and 15% respectively. iCATHe was 65% more successful (95% CI 27% to 75%) at predicting the placement of the return cannula. There were no complications related to the ICE or iCATHe imaging.

**Conclusion:**

iCATHe is a safe and feasible imaging technique to guide real-time VV ECMO cannulae placement and improves accuracy of return cannula positioning compared to ICE.

**Electronic supplementary material:**

The online version of this article (doi:10.1186/2197-425X-2-2) contains supplementary material, which is available to authorized users.

## Background

Extracorporeal membrane oxygenation (ECMO) is a highly specialised form of advanced life support that can be utilised in critically ill patients who require short-term respiratory and/or cardiac support [1–3]. Whilst there are numerous access and cannulation options available for ECMO, they can be classified into two groups: central access and peripheral access [4, 5]. Transthoracic and transoesophageal echocardiography play a fundamental role in the management of patients supported with mechanical support devices, including ECMO [6–10]. Malpositioning of access and return cannulae or inflow/outflow cannulae of any form of mechanical support can have significant adverse consequences. It may cause ineffective delivery of haemodynamic support, haemolysis, increase the risk of ‘suckdown’, acute pulmonary oedema or cardiac trauma. Imaging guidance is important during cannulae insertion and optimal positioning of mechanical cardiac support devices [11, 12] and especially for peripheral cannulae for VV ECMO. Correct access and return cannulae placement in this form of support is required to prevent recirculation and optimise oxygenation.

In contrast to the clinical setting, VV ECMO cannulae guidance using transthoracic and transoesophageal echocardiography in sheep can be challenging, related to certain spatio-anatomic limitations. Intracardiac echocardiography, due to its high spatial resolution and location of the beam former within the right heart [13, 14], has the potential ability to address these limitations. However, during intracardiac echocardiographic assessment of cannulae positioning, it can be difficult to visualise both cannulae clearly due to an echocardiographic reverberation artefact from the initial cannula already in place and from the pulmonary artery catheter *in situ*. This limits accurate assessment of cannulae position. The aim of this study was to assess the feasibility of a novel imaging technique, intracatheter echocardiography (iCATHe), with conventional intracardiac echocardiography (ICE) to guide the placement of both cannulae in an ovine VV ECMO model using post mortem cannulae position as the reference standard.

## Methods

Following animal ethics approval from the University Animal Ethics Committee of the Queensland University of Technology (approval no. 110000053), echocardiographic imaging was performed in our validated VV ECMO ovine model. The investigation conformed to the National Health and Medical Research Council (NHMRC) Code of Practice for the Care and Use of Animals for Scientific Purposes [15]. Anaesthetised sheep (18-month-old ewes, weighing 40 to 45 kg) were commenced on VV ECMO via access (22 F) and return (19 F) cannulae inserted in their capacious right internal jugular vein (IJV). Anaesthesia was induced using intravenous midazolam (0.5 mg/kg) and alfaxalone (3 mg/kg). No muscle relaxants were used. Anaesthesia was maintained with infusions of alfaxalone (4 to 6 mg/kg/h), midazolam (0.25 to 0.5 mg/kg/h), and ketamine (3 to 5 mg/kg/h). An intravenous bolus of buprenorphine 0.01 mg/kg was given for analgesia and subsequently every 6 h. For an extensive discussion regarding the development and management of this ovine ECMO model used in this study, readers are referred elsewhere [16].

The optimal position of the access cannula was in the inferior vena cava just below the diaphragm. A guidewire was inserted into the inferior vena cava using ICE guidance prior to insertion of the access cannula. ICE was performed to guide placement of the initial access cannula, over this guidewire, using a 10-F AcuNav™ probe and Siemens Sequoia™ scanner (Siemens AG, Erlangen, Germany), via an ipsilateral 11 F IJV sheath. The location of the cannula tip was documented. Following the positioning of the access cannula, the ICE probe was withdrawn from the IJV sheath and then passed down inside the actual ECMO cannula (which was fluid-filled to the elevated end and had a cap with a small central lumen sufficient for passage of the ICE probe only) prior to connection to the circuit, noting the imaging of the distinct ECMO cannula tip and then the anatomic location of the tip relative to surrounding cardiac structures. As the ICE probe was 10 F in size and the ECMO cannulae 19 and 21 F in sizes, there were no issues related to insertion, manipulation or withdrawal of the ICE probe within the ECMO cannulae.

The return cannula was then inserted (over a guidewire) into the right atrium using ICE as a guide to optimal positioning, considered as in the high right atrium or right atrial-superior vena cava junction. Following the positioning of the return cannula, the ICE probe was again passed down inside the return cannula prior to connection to the circuit, noting the location of the cannula tip relative to the surrounding cardiac structures. If the cannula was not visualised with ICE but seen with iCATHe imaging and identified to be incorrectly positioned, this was noted and then manipulated to a better location, using iCATHe guidance. The cannulae were secured using cyanoacrylate adhesive and intracutaneous stay sutures.

**Figure 1:**
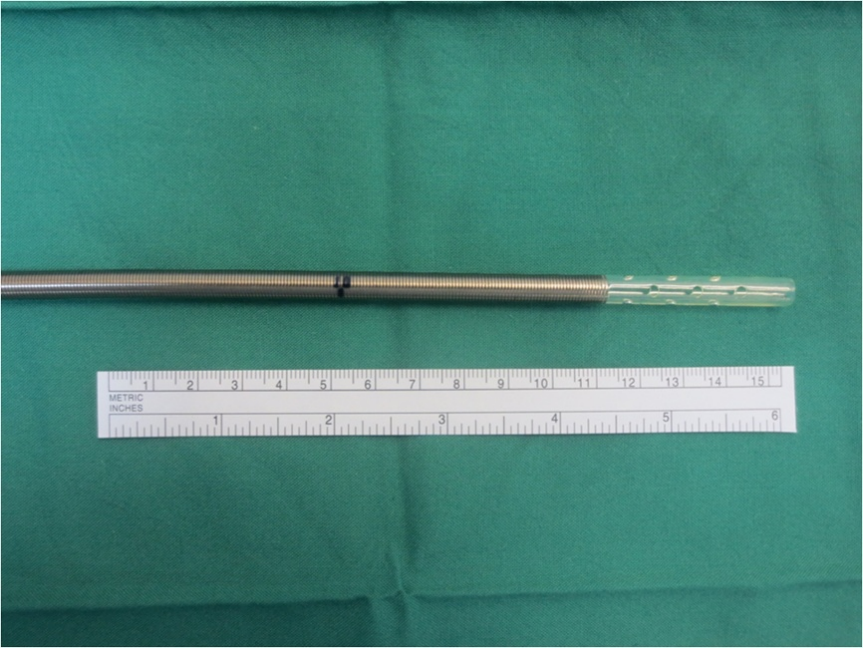
**19 F ECMO cannula.** Note the main body of the cannula with circumferential metal wiring for radial structural support and the distal tip composed of polyurethane with multiple side holes and a main central lumen at the end.

**Figure 2:**
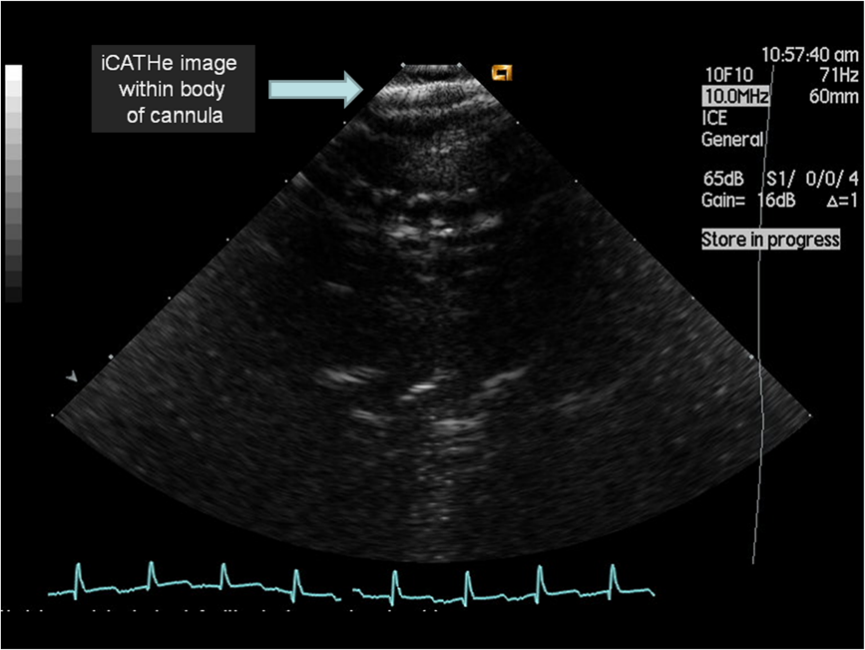
**iCATHe image acquired whilst within the metallic component of the cannula.** Note the continuous linear metal reflective signal.

**Figure 3:**
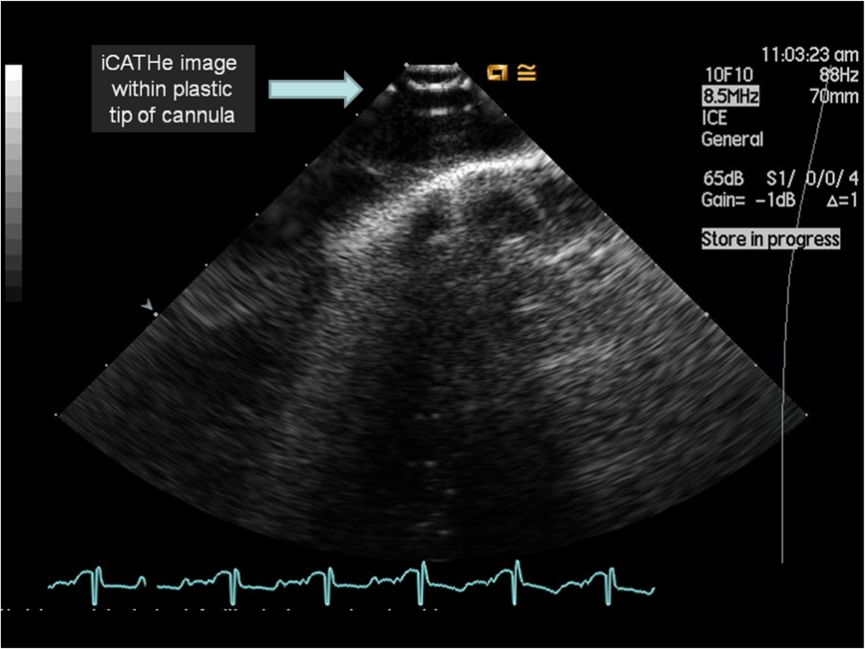
**iCATHe image acquired whilst within the polyurethane tip.** Note the perfusion/flow side holes.

**Figure 4:**
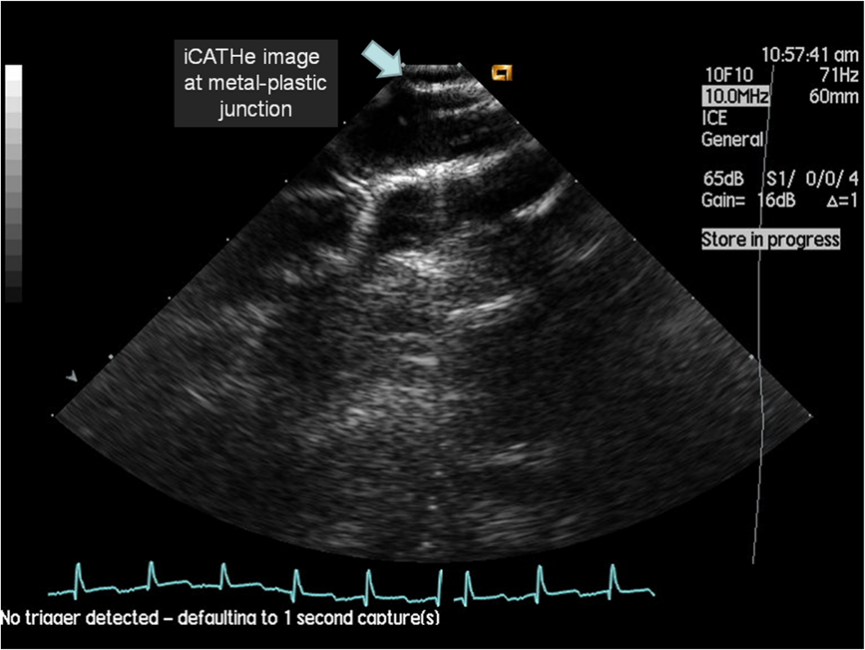
**iCATHe image of an access cannula within the inferior vena cava.** Note the transition from the metallic component of the cannula (right side of image) to the polyurethane tip of the cannula (left side of the image, with a side hole on view).

**Figure 5:**
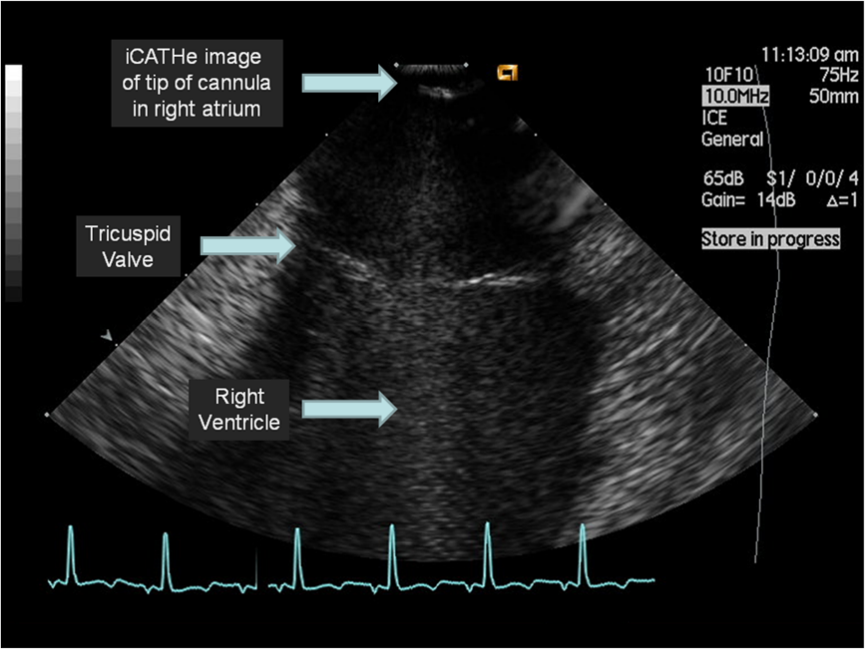
**An iCATHe image as the tip of the ICE probe just exits out the end of the ECMO cannula.** Note the clear visualisation of the tricuspid vale, right ventricle and end of the ECMO cannula.

In light of the novel images obtained by passing an ICE probe down an ECMO cannula, a water phantom model was developed to determine how the echocardiographic image would appear from within an ECMO cannula. Figure 1 shows an ECMO cannula *ex vivo*. The majority of the length of the ECMO cannula has circumferential, flexible metal reinforcement, enhancing radial strength to help minimise cannula kinking. This reinforced section had a distinct metallic reflective pattern during ICE imaging (Figure 2). Once within the distal polyurethane tip of the cannula (the final 4 cm), there was a softer acoustic reflective pattern seen along with clear visualisation of the numerous side holes (Figure 3). Figure 4 depicts the transition from metallic to polyurethane construction of the ECMO cannula, as imaged by an ICE probe within the cannula. Finally, as the ICE catheter passed out the end of the ECMO cannula, a more conventional ICE image of the right heart was obtained. Figure 5 depicts an ICE image with the tip just out the end of the ECMO cannula, which can be seen to the right of the image. Video S1 in Additional file 1 shows the withdrawal of the ICE probe from the inferior vena cava back into the ECMO cannula. Video S2 in Additional file 2 shows passage of the ICE probe down through the ECMO cannula and out the end into the right atrium.

Following cannulae placement, VV ECMO was commenced and continued for 24 h. After 24 h, the sheep were euthanized and cannulae position was determined at post mortem. Echocardiographic variables analysed were the ability to image the access and return cannulae tip, location of the cannulae tip and presence of any air within the right heart during cannulation. The two-tailed McNemar test (Medcalc®, Ostend, Belgium) was used to compare iCATHe with ICE cannulae positioning, with the reference standard being the location determined at post mortem.

## Results

**Figure 6:**
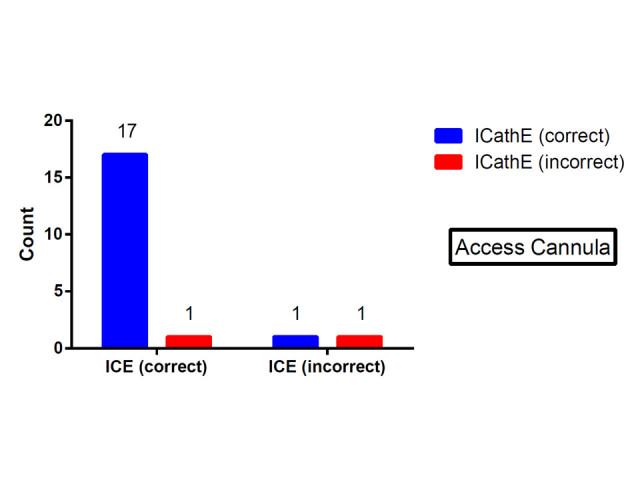
**ICE versus iCATHe results for the access cannula.**

**Figure 7:**
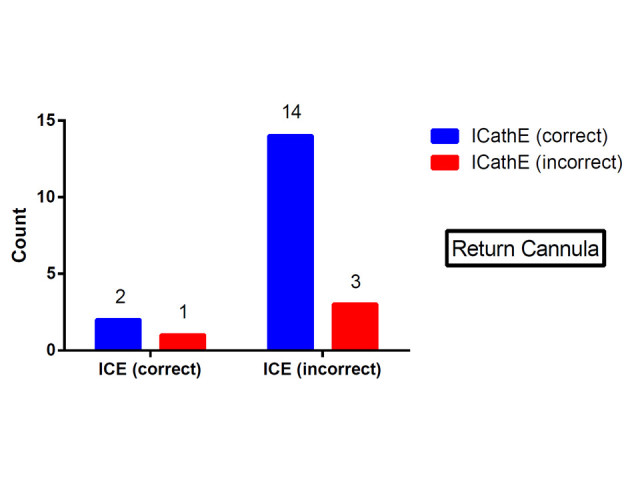
**ICE versus iCATHe results for the return cannula.**

Cannula positioning in 20 sheep on VV ECMO was assessed using both ICE and iCATHe. The guidewire for both the access and return cannulae insertion was visualised in all cases. ICE and iCATHe imaging was technically possible in all cases. Table 1 represents the McNemar tabulations for the access and return cannulae positioning (ICE versus iCATHe). Figure 6 depicts the ICE versus iCATHe result for the access cannula. Figure 7 depicts the ICE versus iCATHe result for the return cannula. There was no significant difference between the two methods in assessing the access cannula position (proportion correct for each 90%, incorrect 10%).

**Table 1:** **McNemar tabulations for the access and return cannulae positioning (ICE versus iCATHe)**

Access cannula		ICE		
		Correct	Incorrect	Total
iCATHe	Correct	17	1	18
	Incorrect	1	1	2
	Total	18	2	20
**Return cannula**		**ICE**		
		**Correct**	**Incorrect**	**Total**
iCATHe	Correct	2	14	16
	Incorrect	1	3	4
	Total	3	17	20

However, there was a significant difference between ICE and iCATHe success rates for the return cannula position (*p* = 0.001). The proportion correct for iCATHe and ICE was 80% and 15% respectively. iCATHe was 65% more successful (95% CI 27% to 75%) at predicting the placement of the return cannula.

There were no complications related to the ICE or iCATHe imaging. There was no entrainment of air into the circuit or the heart during any of the procedures. There was no loss of circulating volume from the ‘open’-ended cannulae during the iCATHe imaging.

## Discussion

ECMO is a form of extracorporeal life support (ECLS) that is used to treat refractory respiratory and/or cardiac failure. The mechanism of action relies on gas exchange (carbon dioxide removal, oxygenation) and hemodynamic support, which is mediated via blood flow between the ECMO circuit and native circulation using large bore cannulae [17]. The accurate positioning of these cannulae is paramount for effective delivery of ECMO support. Echocardiography plays a key role in facilitating this [6] and in humans, this takes the form of transthoracic and transoesophageal echocardiography [18]. To date, there have been no published studies assessing the utility of ICE in guiding ECMO in clinical setting.

In this study which used an ovine model, two different forms of echocardiographic imaging were performed to determine cannula positioning, standard ICE and iCATHe. Conventional transthoracic and transesophageal echocardiographic imaging in animal models can be challenging. Open-chested epicardial imaging can overcome this [19–21], but with the significant limitation of its invasiveness. More recently, ICE imaging has been used in animal models [22–24]. ICE was chosen as the conventional form of imaging to guide cannulae placement due to its ready availability, high spatial resolution and lack of a suitable alternative echocardiographic technique. Unlike in humans, transthoracic echocardiography in sheep can be technically difficult due to the small acoustic window often present and the shape of the chest wall, with modified parasternal long and short axis views being the best reproducible images, neither of which would have helped significantly with guiding cannula placement. Modified transoesophageal echocardiography is feasible in sheep [25] but it may not provide consistent quality imaging due to the capacious nature of an ovine oesophagus, limiting probe contact required to regularly generate satisfactory images.

Conventional ICE imaging provided reliable and consistent imaging of the superior vena cava, right heart and inferior vena cava. As such, ICE was used as the reference standard to assess the feasibility of iCATHe imaging. Location of the guidewire was possible in all cases, with confirmation of the wire in the inferior vena cava routinely performed. ICE could also detect if the wire had prolapsed into the right ventricle. This was important because during insertion of the introducer/cannula over the wire, displacement of the wire from its original appropriate position in the inferior vena cava (IVC) could and did occur. Without recognition of this via ICE imaging, the access cannula could have been positioned within the right ventricle. This has the dual adverse effects of recirculation and increased risk of cardiac trauma or perforation. Whenever the wire was displaced into the right ventricle, the cannula was withdrawn and the guidewire was reinserted into the inferior vena cava using ICE guidance. ICE could also detect any thrombus formation on the guidewire or cannula during insertion and manipulation.

In our study, the access cannula was always inserted first, followed then by the return cannula. This was done to minimise the impact of any possible cannula co-dependence, in the light of both cannulae being inserted into the same jugular vein. If the return cannula was placed first within the right atrium, subsequent insertion and manipulation of the access cannula may have resulted in displacement of the return cannula, where there is reduced margin for error for altered positioning. Additionally, prior to any ECMO cannulae insertion, a pulmonary artery catheter was inserted via an internal jugular vein route. It was the presence of this pulmonary artery catheter and the already positioned access cannula which made it difficult to use ICE to image the return cannula. As the ICE probe was in close proximity to the pulmonary artery catheter and access cannula, it was difficult to obtain ICE images to manipulate and position the return cannula. This was primarily due to reverberation artefact from the access cannula. Video S3 in Additional file 3 is an example of ICE imaging during return cannula positioning, displaying this difficulty. By utilising iCATHe imaging, it was possible in most cases to have a greater spatial awareness of the location of the tip of the return cannula. This was particularly the case when the ICE probe was within the polyurethane tip and then exited the cannula, where ICE probe manipulation just beyond the tip could determine whether the cannula was in the superior vena cava or the right atrium.Additional file 3: Video S3: ICE of the middle right atrium during return cannula insertion. Note the difficulty in determining cannula tip location. (AVI 4 MB)

iCATHe imaging did not offer any advantage over conventional ICE imaging for placement of the access cannula. This could be anticipated as ICE provided a clear imaging of the wire and then cannula positioning in the inferior vena cava. Consequently, additional alternative imaging would not be expected of any incremental benefit. However, due to the limited ICE imaging obtained to view the return cannula, as outlined above, the addition of iCATHe imaging provided a clearer alternative to determine return cannulae placement.

Despite the iCATHe imaging occurring as an open procedure with communication of the circulation with the atmosphere via the ECMO cannulae, there were no air embolisation events or bleeding from the cannula. Blood loss from the cannula end did not occur, as both cannulae were venous and the external end was raised slightly to prevent retrograde flow of blood. Additionally, any possible entrainment of air was countered by having the cannula fluid-filled, elevating the external tip and placing a cap on the end, with a small aperture which would allow passage of the ICE probe only.

Echocardiography plays an important role in positioning of peripherally inserted ECMO cannulae, especially for VV ECMO in respiratory failure. To January 2013, there were 53,190 cases of ECMO listed on the Extra Corporeal Life Support (ELSO) registry [26]. Of these, 35,622 (67%) were for a respiratory indication. Correct location and orientation between the two VV ECMO cannulae are required to prevent a phenomenon called ‘recirculation’. Recirculation occurs if the VV ECMO access and return cannulae are too close to one another or if the access cannula is located more proximal than the return cannula. Recirculation will then occur, with the oxygenated blood being returned straight back into the circuit and not being delivered systemically to the patient. Additionally, if cannulae are positioned incorrectly, this and the associated manipulation or surgery to reposition them can increase the risk of infection, bleeding, cardiac trauma or sub-optimal flows [27–29].

Imaging to guide and evaluate ECMO cannulae positioning has been studied in the neonatal and paediatric population. These studies indicated that transthoracic echocardiography can be utilised to reposition cannulae [30], enhance accuracy of cannulae positioning at insertion [31] and was more accurate than chest x-ray (CXR) in determining cannula positioning [32, 33]. CXR to guide and assess cannulae location has numerous potential advantages, such as it requiring little specific operator experience, making it readily available and relatively economical [34]. However, limitations of using a CXR to guide cannulae placement include that many ECMO cannulae do not have a radio-opaque tip and exposure of staff to ionising radiation. Additionally, unless fluoroscopy is used during actual insertion, it cannot offer real-time feedback on cannula manipulation to optimise placement.

**Figure 8:**
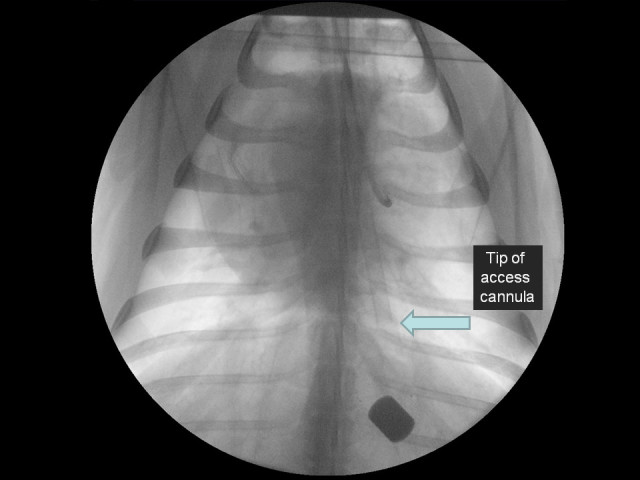
**Plain chest x-ray of a sheep during VV ECMO.** Note the visualisation of only the access cannula within the inferior vena cava.

In our study, we did not routinely use CXR to assess cannula positioning. Figure 8 depicts a CXR of a sheep on VV ECMO. The thoracic anatomy of sheep, with apical ‘crowding’ of structures, reduced accurate visualisation of the return cannula. The arrow points to the end of the radio-opaque tip of the access cannula, just above the diagram. The actual cannula extends a further 4 cm beyond this, as it is constructed with polyurethane and hence not visualised using a CXR. In light of the exposure to ionising radiation and the inability to directly visualise the tip of the ECMO cannulae, fluoroscopy was deemed as unsuitable as a gold standard in our study. Additionally, in a clinical context within the critical care complex, where ECMO is often initiated, fluoroscopy is limited by sub-optimal image quality and the requirement to protect a significant number of staff members from ionising radiation.

Imaging is fundamental for insertion of the bicaval, dual-lumen cannula for VV ECMO support. This single but dual-purpose cannula drains blood from proximal and distal ports within the superior and inferior vena cava, respectively. The second central lumen then returns blood back to the right heart where it exits the cannula from a central port directed toward the tricuspid valve [27]. This dual-lumen cannula option offers the advantage of a single cannulation site, enhancing the likelihood of patient mobilisation and reducing the likelihood of recirculation. However, in light of its triple-orifice design, meticulous positioning is necessary and insertion and manipulation should be done using appropriate imaging to confirm that all three ports are in the correct location [35–37]. This imaging is often performed by transoesophageal echocardiography [35, 38]. Transthoracic echocardiography with or without using agitated saline injections has also been used to help determine positioning [39–41]. iCATHe may offer an alternative imaging modality to determine location of larger dual-lumen cannulae if there is a contraindication to transoesophageal echocardiography or if transthoracic echocardiographic image quality results in a non-diagnostic study.

### Study limitations

As this was a novel echocardiographic imaging technique, just one operator (DP) performed all the scanning to achieve an adequate skill and knowledge base for this technique. Hence, the widespread applicability and feasibility of this technique cannot be assessed from this study. However, in light of the reported relatively quick learning curve for this technique, it is likely that any operator with appropriate echocardiographic skills, who is involved in guidance for ECMO initiation, would be able to perform iCATHe.

Cannula placement using the two echocardiographic techniques was compared to post mortem analysis, which was 24 h after placement. Hence, it is possible that cannula may have been inadvertently displaced during this 24-h period or during the post mortem process. Whilst no sitting marks were placed on the cannulae following insertion to counter this, the cannulae were firmly glued and sutured in place and care was taking during the post mortem to minimise any possible cannula displacement.

The cannulation methodology of this animal ECMO model has two differences to that of human ECMO. Firstly, in humans, the jugular vein approach for access and return cannulae, a pulmonary artery catheter (PAC) and an ICE probe would usually not be used. Secondly, this study was performed in an animal model with a PAC *in situ* to enable monitoring of systemic and pulmonary haemodynamic parameters. Whilst in the clinical setting there is variation in the use of a PAC in the critical care complex, they may not be utilised in patients supported with VV ECMO. As such, the imaging of the respective ECMO cannulae may have been improved in the absence of a PAC causing acoustic shadowing.

This iCATHe technique was performed in an ovine ECMO model under controlled experimental conditions. The question remains: can this research be translated to the human clinical environment? Whilst the results of this feasibility study suggest that assessment of return cannulae positioning is significantly improved using iCATHe compared to ICE, there would be several clinical barriers that would limit or prevent clinical introduction of this technique. A major limitation to this would be the perceived risk of having an ‘open-ended’ cannula, potentially exposing the patient's circulation to air entrainment or significant bleeding. However, utilising the preventative measures employed in this study ensured that neither of these two complications occurred. Compounding this would be the requirement for meticulous sterility techniques to minimise any infection risk with an open technique. In our ovine study, no data was collected involving microbiological cultures or any control group used, so no conclusions can be drawn regarding the risk of systemic infection by using the iCATHe technique. Additionally, there may be a significant economic cost of intracardiac echocardiographic probes, and in some countries or institutions, as these may be single-use only, further increasing the cost of this technique.

This technique could have a role in a small sub-group of patients who are VV ECMO candidates, where there is a concern regarding cannulae placement and in which conventional imaging (such as transoesophageal echocardiography) has not been able to satisfactorily guide cannulae placement. An additional potential benefit of iCATHe imaging in humans is that it neither requires any additional venous access or puncture nor oesophageal intubation. The imaging probe is passed down inside the already inserted cannula and this could then be manipulated to the correct position using real-time guidance from the ultrasound image. In this regard, iCATHe may have a role insertion of a dual-lumen cannula. The ICE probes are typically 90 cm in length, which is sufficient to image the right side of the heart via a femoral approach. However, iCATHe may not provide further detailed global assessment of the cardiac structure and function, as compared to transoesophageal echocardiography, which is often of relevance to clinical care. Additionally, iCATHe is also of no benefit in assessing cannulae position once ECMO has commenced, as the circuit is closed and the cannulae are inaccessible.

## Conclusions

Echocardiography plays a fundamental role in guiding cannulae insertion during initiation of VV ECMO. We report a novel echocardiographic imaging technique, iCATHe, as being a safe and feasible imaging technique to guide real-time VV ECMO cannulae placement and improves accuracy of return cannulae positioning compared to ICE in an ovine model. Further safety and efficacy assessment of the iCATHe technique is required. However, it has the potential to be utilised in other large animal models and in a small subset of human patients on VV ECMO.

## Electronic supplementary material

Additional file 1: Video S1: iCATHe movie of an access cannula within the inferior vena cava. The ICE probe is withdrawn back from within the IVC, passing through the polyurethane tip and finishes within the metal component of the cannula. (MPEG 2 MB)

Additional file 2: Video S2: iCATHe return cannula within the right atrium. The ICE probe is advanced through the catheter, initially from within the metallic component, through the polyurethane tip and out of the end of the cannula and imaging the central right atrium down to the tricuspid valve and right ventricle. (MPEG 1 MB)
